# The GS-nitroxide JP4-039 improves intestinal barrier and stem cell recovery in irradiated mice

**DOI:** 10.1038/s41598-018-20370-9

**Published:** 2018-02-01

**Authors:** Liang Wei, Brian J. Leibowitz, Michael Epperly, Cheng Bi, Allen Li, Justin Steinman, Peter Wipf, Song Li, Lin Zhang, Joel Greenberger, Jian Yu

**Affiliations:** 1 Department of Pathology, University of Pittsburgh School of Medicine, UPMC Hillman Cancer Center, Pittsburgh, PA 15213 USA; 2 Department of Radiation Oncology, UPMC Hillman Cancer Center, Pittsburgh, PA 15213 USA; 3 Department of Bioengineering, University of Pittsburgh, Swanson School of Engineering, Pittsburgh, USA; 4 Department of Medicine, University of Pittsburgh, Pittsburgh, PA 15260 USA; 5 Department of Chemistry, University of Pittsburgh, Pittsburgh, PA 15260 USA; 6 Department of Pharmaceutical Sciences, University of Pittsburgh, Pittsburgh, PA 15260 USA; 7 Department of Pharmacology and Chemical Biology, University of Pittsburgh School of Medicine, Pittsburgh, PA 15213 USA

## Abstract

Total body irradiation (TBI) leads to dose- and tissue-specific lethality. In the current study, we demonstrate that a mitochondrion-targeted nitroxide JP4-039 given once 24 hours after 9–10 Gy TBI significantly improves mouse survival, and the recovery of intestinal barrier, differentiation and stem cell functions. The GI-protective effects are associated with rapid and selective induction of tight junction proteins and cytokines including TGF-β, IL-10, IL-17a, IL-22 and Notch signaling long before bone marrow depletion. However, no change was observed in crypt death or the expression of prototypic pro-inflammatory cytokines such as TNF-α, IL-6 or IL-1β. Surprisingly, bone marrow transplantation (BMT) performed 24 hours after TBI improves intestinal barrier and stem cell recovery with induction of IL-10, IL-17a, IL-22, and Notch signaling. Further, BMT-rescued TBI survivors display increased intestinal permeability, impaired ISC function and proliferation, but not obvious intestinal inflammation or increased epithelial death. These findings identify intestinal epithelium as a novel target of radiation mitigation, and potential strategies to enhance ISC recovery and regeneration after accidental or medical exposures.

## Introduction

The intestinal epithelium is the fastest renewing tissue in an adult mammal, and carries out vital functions such as nutrient and water absorption, neurotransmitter secretion, and serving as a physical barrier against microorganisms. The intestinal epithelium is under continuous mechanical and pathogenic insults, and is replenished with a cycle estimated to be 3–5 days in mice^1–3^. Intestinal stem cells (ISCs) are located in the bottom of crypts, and produce progenitor and transit amplifying cells that differentiate into four major epithelial cell types including enterocytes, goblet cells, enteroendocrine cells, and Paneth cells, while mature cells are lost via apoptosis from the villus tip^4–6^. Wnt, BMP, EGF, and Notch regulate intestinal proliferation and differentiation during homeostasis, involving complex crosstalk of different cell types including Paneth cells, subepithelial myofibroblasts, endothelial cells, and enteric neurons^7–9^. Additionally, immune cell-derived IL-22 was recently shown to regulate ISC regeneration after injury^10^.

The gastrointestinal (GI) tract closely interacts with 80% of the immune system and microorganisms that outnumber the host cells by a factor of ten, a complex interaction regulated by the epithelial barrier that is compromised in most, if not all forms of GI injury^11,12^. The intestinal barrier is governed by glycoproteins such as Goblet cell-secreted mucin, as well as complexes containing adherens junction (AJ) and tight junction (TJ) proteins that lock adjacent cells together. These complexes have been shown to regulate cellular polarization, proliferation, and differentiation^12^, and are also regulated by T cell-produced IL-17a in healthy mice^13,14^. The barrier is believed to play a key role in intestinal injury and regeneration through local and systemic effects, but little is known about what regulates the recovery of intestinal barrier and ISCs and if it is coordinated.

Radiation is known to cause dose- and tissue-specific acute injury and lethality correlated with the tissue stem cell renewal cycles^15^. Radiation causes DNA strand breaks and oxidative damage, stalled or collapsed replication forks, as well as damage to other macromolecules^16^. At higher doses (i.e. 14–18 Gy), TBI causes lethal GI injury and GI syndrome within 7–10 days in mice^15,17^ due to depletion of Lgr5+ stem cells^18–20^, which is controlled by the p53 pathway and DNA repair proteins^15,20–22^. At lower doses (6–10 Gy), TBI causes lethal hematopoietic (HP) syndrome within 2–4 weeks, which can be rescued by bone marrow transplantation (BMT)^15,17^. In fact, transplantation of BM stromal cells also improves the survival of irradiated mice, which is associated with a systemic increase of intestinal trophic factors such as basic fibroblast growth factor (bFGF) and R-Spondin1, and restoration of the intestinal barrier^21,23–26^.

Nitroxides are antioxidants that catalyze the dismutation of superoxide radicals^27^. Our group developed JP4-039 as a mitochondrion-targeted derivative of 4-amino-Tempo (4-AT), which effectively protects and mitigates TBI-induced HP syndrome, and accumulates in multiple tissues including the intestine^28,29^. In the current study, we found that TBI induces acute and delayed intestinal barrier and stem cell dysfunction. A single administration of JP4-039 24 hours after TBI improves mouse survival and intestinal recovery without affecting cell death. Surprisingly, BMT performed 24 hours after TBI also improves the intestinal recovery from barrier and stem cell dysfunction. Our study identifies intestinal epithelium as a novel target of radiation mitigation, and potential strategies to enhance ISC recovery and regeneration after accidental or medical exposures^30,31^.

## Results

### JP4-039 given 24 hours after TBI prolongs survival and mitigates impaired intestinal differentiation

**Figure 1:**
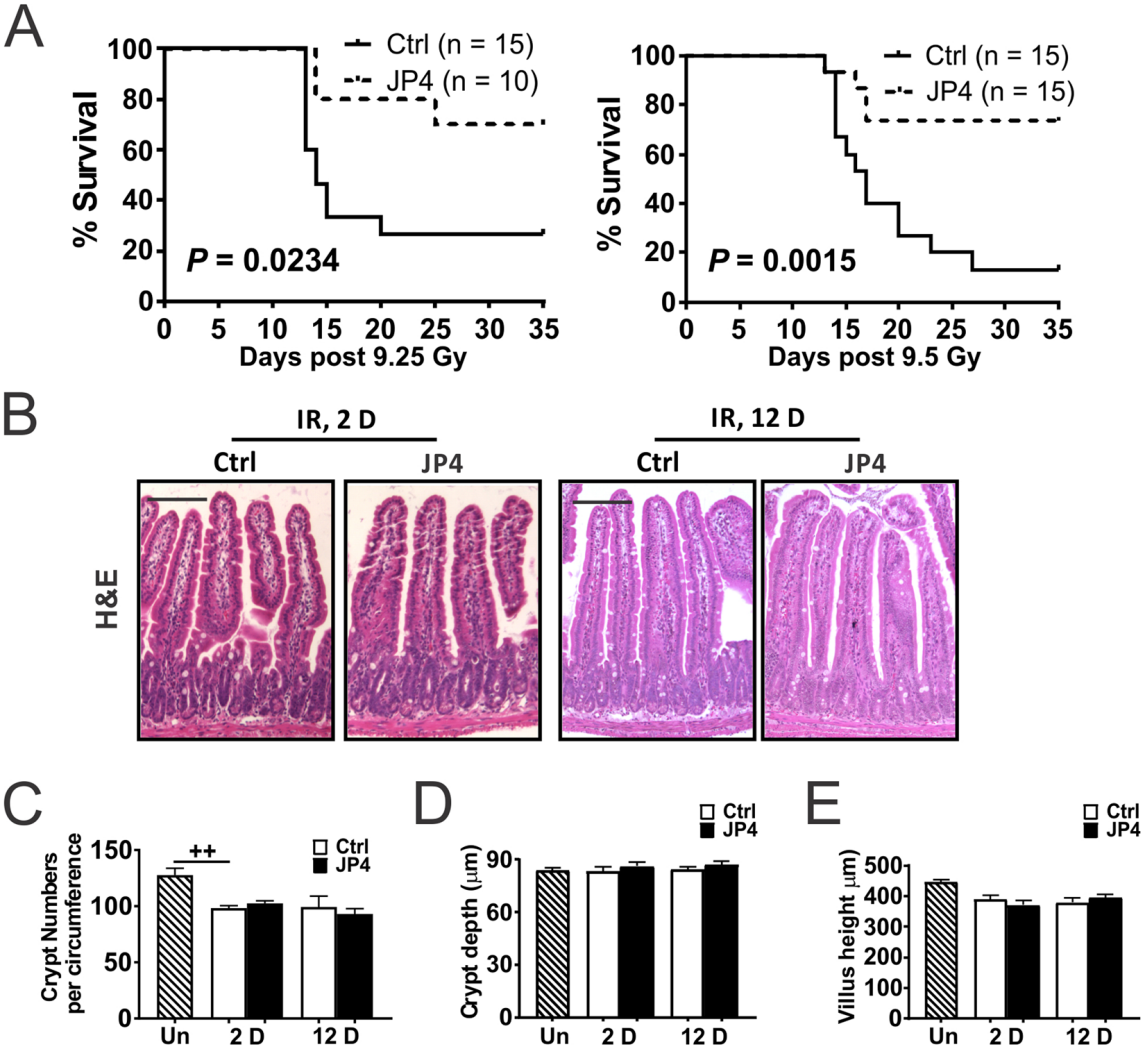
JP4-039 given once 24 hours post TBI prolongs survival in mice. (**A**) Kaplan-Meier survival curves of mice subjected to 9.25 and 9.5 Gy TBI. JP4-039 (JP4, 20 or 5 mg/kg in F14 emulsion) or vehicle (Ctrl) was given to mice once 24 hr (Day 1) after TBI. *P* = 0.0234 and 0.0015 respectively, by Log-Rank test. (**B**) Representative H&E staining of intestinal sections on Day 2 and 12 after 9.25 Gy TBI. Scale bar = 100 µm. (**C**) Quantification of crypt numbers, (**D**) crypt depth, and (**E**) villus length in B. (**C**,**D**,**E**) values are Mean ± SEM; n = 3 mice in each group. ^++^*P* < 0.01, by 1-way ANOVA followed by Tukey’s multiple comparisons test.

Consistent with earlier reports^28,29^, intravenous (IV) administration of JP4-039 once by 24 hours after 9.25 Gy or 9.5 Gy total body irradiation (TBI) significantly improved survival of mice from 25%, or 10% to 75% by Day 35. Death starting around 2 weeks is characteristic of lethal HP syndrome (Fig. 1A). To determine if intestinal epithelium is a target of mitigation, we examined the histology on Day 2 and Day 12 after 9.25 Gy TBI. TBI induced a minor decrease in crypt numbers and villi length with little or no change in structure, completely lacking the enlarged “regenerated crypts” from single clonogenic stem cells seen after high-dose radiation. These structural features were indistinguishable between the control and JP4-039 groups (Figs 1B–E and S1).

**Figure 2:**
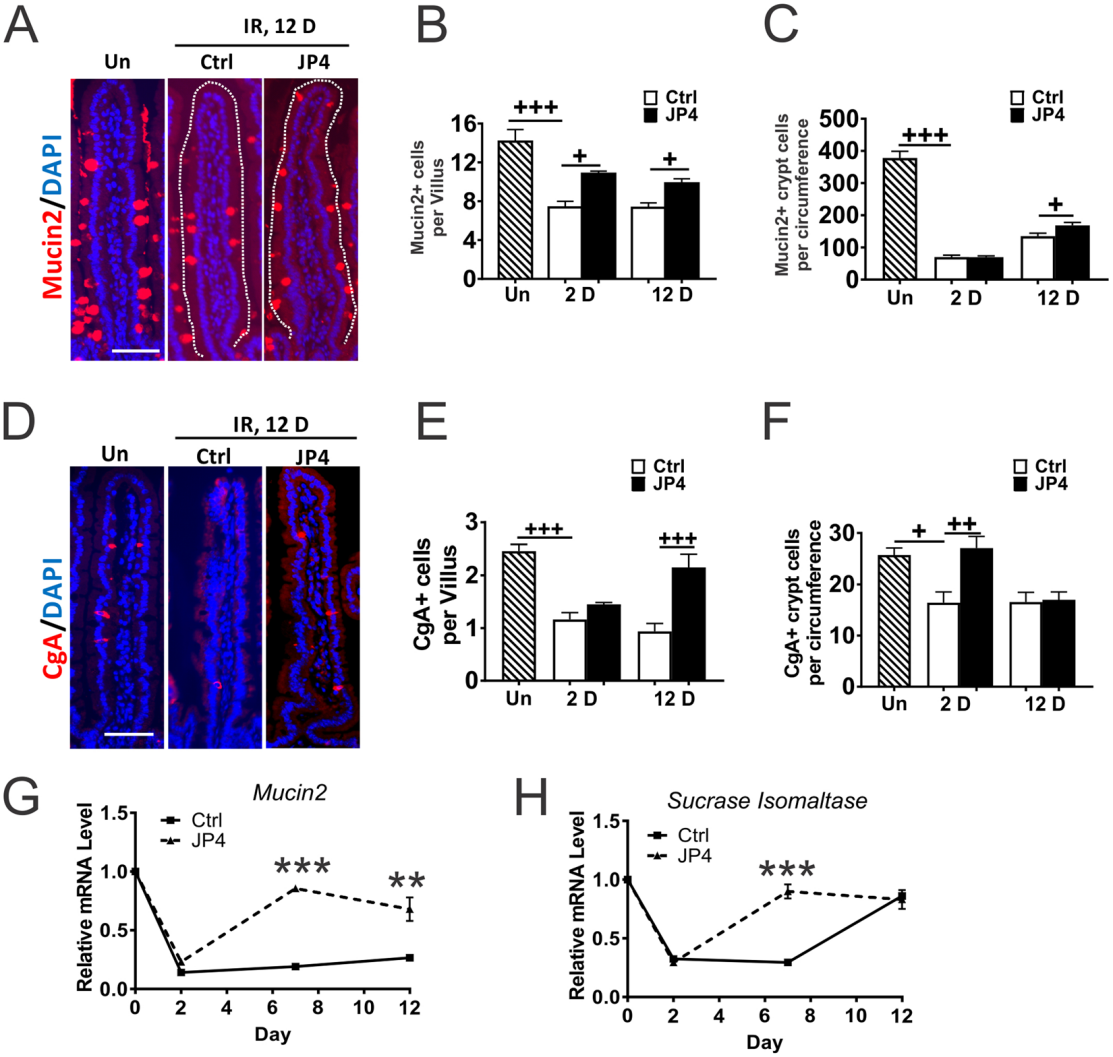
JP4-039 suppresses TBI-induced intestinal differentiation defects. JP4-039 (JP4, 20 mg/kg in F14 emulsion) or vehicle (Ctrl) was given to mice once 24 hr (Day 1) after 9.25 Gy TBI. Intestinal tissues were analyzed at indicated times after TBI. (**A**) Representative immunofluorescence staining of Mucin2 in the villi. Red, Mucin2; Blue, DAPI. Scale bar = 100 µm. (**B**) Quantification of Mucin2+ cells in villi and (**C**) in the crypts from A. (**D**) Representative immunofluorescence staining of Chromogranin A+ (CgA) cells in the villi. Red, CgA; Blue, DAPI. Scale bar = 100 µm. (**E**) Quantification of CgA+ cells in the villi and (**F**) in the crypts from D. (**G**) Mucosal mRNA expression of *Mucin2* and (**H**) *Sucrase lsomaltase*. cDNA was synthesized from RNA pooled from 3 mice per group. Expression was normalized to that at Day 0, prior to TBI. (**B**,**C**,**E**,**F**,**G** and **H**) values are Mean ± SEM; n = 3 mice in each group. ^+++^*P* < 0.001, ^++^*P* < 0.01, ^+^*P* < 0.05, 1-way ANOVA followed by Tukey’s multiple comparisons test. ***P < 0.001, **P < 0.01, vehicle *vs*. JP4, unpaired 2-tailed Student’s t test.

We then assessed intestinal differentiation at 2 and 12 days after TBI before any casualty. Immunofluorescence staining indicated a 50% reduction of goblet (Mucin2+) and enteroendocrine (Chromogranin A, CgA+) cells in the villi on day 2 after TBI in the control group which persisted to day 12 (Figs 2A–F, S2A–B). In the JP4-039 group, goblet cell loss was reduced by 50% (Figs 2A–C, S2A), and enteroendocrine (CgA+) cell loss nearly fully recovered by day 12 (Figs 2D–F, S2B). These effects were more pronounced in the villi where most differentiated cells are found, compared to the crypts. Neither TBI nor JP4-039 significantly altered the numbers or localization of Paneth (MMP7+) cells, which are part of the Wnt3-producing ISC niche^32^ (Fig. S2C–D). qRT-PCR was used to monitor changes in lineage transcripts, and confirmed a rapid loss of *Mucin 2* and *Sucrose Isomaltase* (enterocyte marker) expression after TBI on Day 2, which recovered significantly faster in the JP4-039 group by Day 12 (Fig. 2G–H). These data indicate that 9.25 Gy TBI impairs intestinal differentiation, which is mitigated by JP4-039.

### JP4-039 given 24 hours after TBI maintains the intestinal barrier

**Figure 3:**
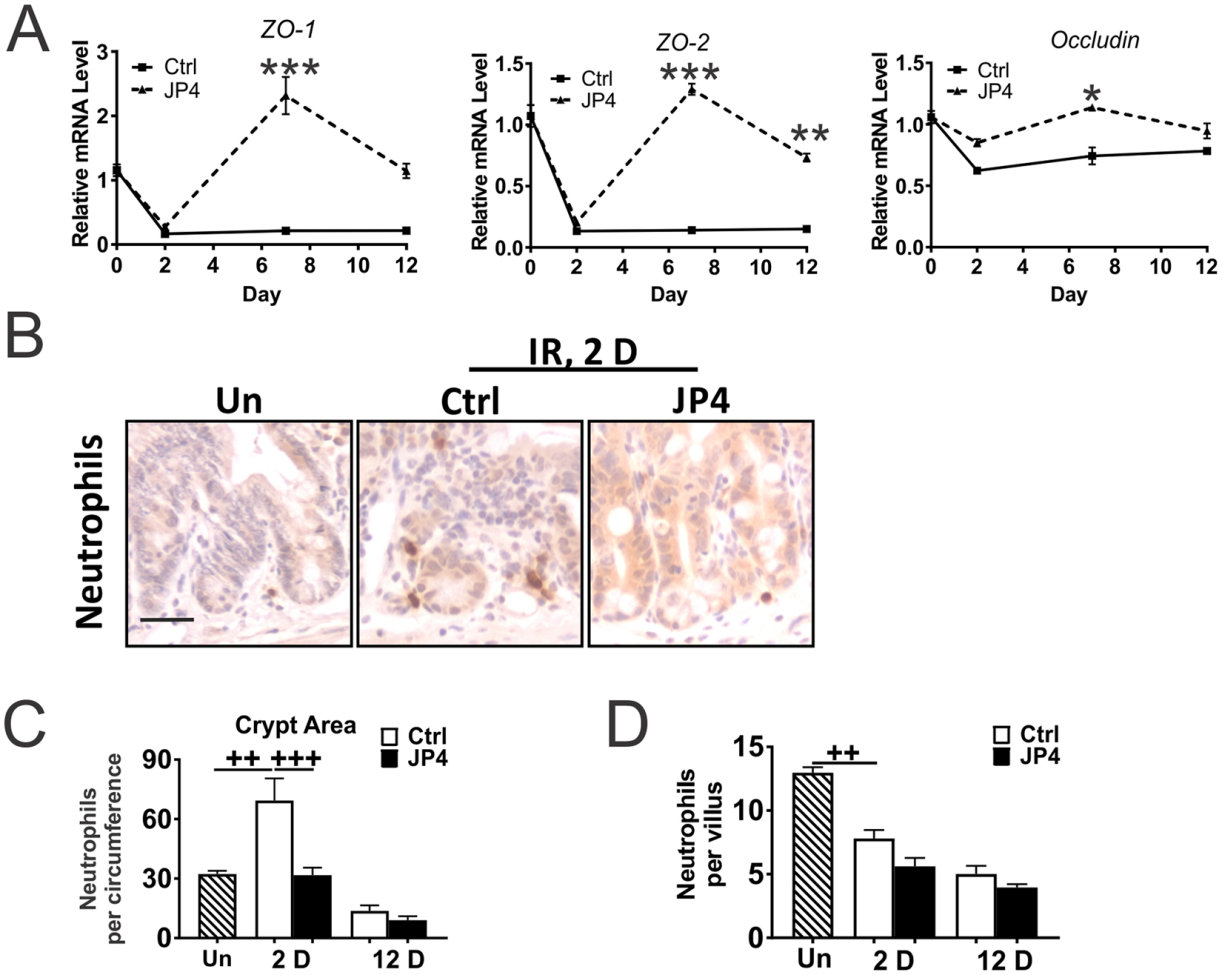
JP4-039 mitigates TBI-induced barrier dysfunction. JP4-039 (JP4, 20 mg/kg in F14 emulsion) or vehicle (Ctrl) was given to mice once 24 hr after 9.25 Gy TBI. Intestinal tissues were analyzed at indicated times (day) after TBI. (**A**) Mucosal expression of *ZO-1*, *ZO-2* and *Occludin* mRNAs. cDNA was synthesized from RNA pooled from 3 mice per group. Expression was normalized to that on Day 0, prior to TBI. (**B**) Representative immunohistochemistry staining of Ly-6B.2 (neutrophils) in the villi. Scale bar = 25 µm. (**C**) Quantification of neutrophils in the crypts and (**D**) villi. (**A**,**C**,**D**) values are Mean ± SEM; n = 3 mice in each group. ***P < 0.001, *P < 0.05, vehicle *vs*. JP4, unpaired 2-tailed Student’s t test. ^+++^*P* < 0.001, ^++^*P* < 0.01, 1-way ANOVA followed by Tukey’s multiple comparisons test.

Severe loss of epithelial structural integrity leads to the GI syndrome after high dose TBI within a week, but was absent in mice recovered from HP syndrome with BMT^15^. Altered expression of TJ proteins such as cadherins, claudins, occludin, and junctional adhesion molecules (JAM) has been shown to destabilize TJs and increase permeability^11,12^. qRT-PCR analysis indicated a rapid and near complete loss of expression of TJ transcripts *ZO-1* and *ZO-2* on Day 2, which persisted through Day 7 and 12. JP4-039 given at 24 hours fully recovered their expression by Day 7 and maintained it through Day 12 (Fig. 3A). Changes in other TJ transcripts such as *Occludin, ZO-3*, *and Claudin-2* were modest on Day 2 in the control group, but returned to basal levels faster on Day 12 in the JP4-039 group (Fig. 3A and S3A). ZO-1 staining confirmed its selective loss in the crypt cells and maintenance in the JP4-039 group on Day 12 (Fig. S3B–D).

Intestinal barrier breakdown causes invasion of microorganisms and recruitment of innate immune cells such as neutrophils^11,12^. Ly-6B.2 staining indicated an over 2-fold and transient increase of neutrophils around the crypts as early as Day 2, which was prevented by JP4-039 (Fig. 3B–C). By Day 12, neutrophils further decreased around the crypts and in the villi, indicative of systemic immunosuppression with BM depletion. JP4-039 had no effect on the numbers of villus neutrophils on Day 2 or 12 (Fig. 3D). These results demonstrate that JP4-039 mitigates intestinal barrier dysfunction and immune infiltrates around the crypts long before bone marrow ablation.

### JP4-039 selectively elevates the expression of intestinal protective cytokines

**Figure 4:**
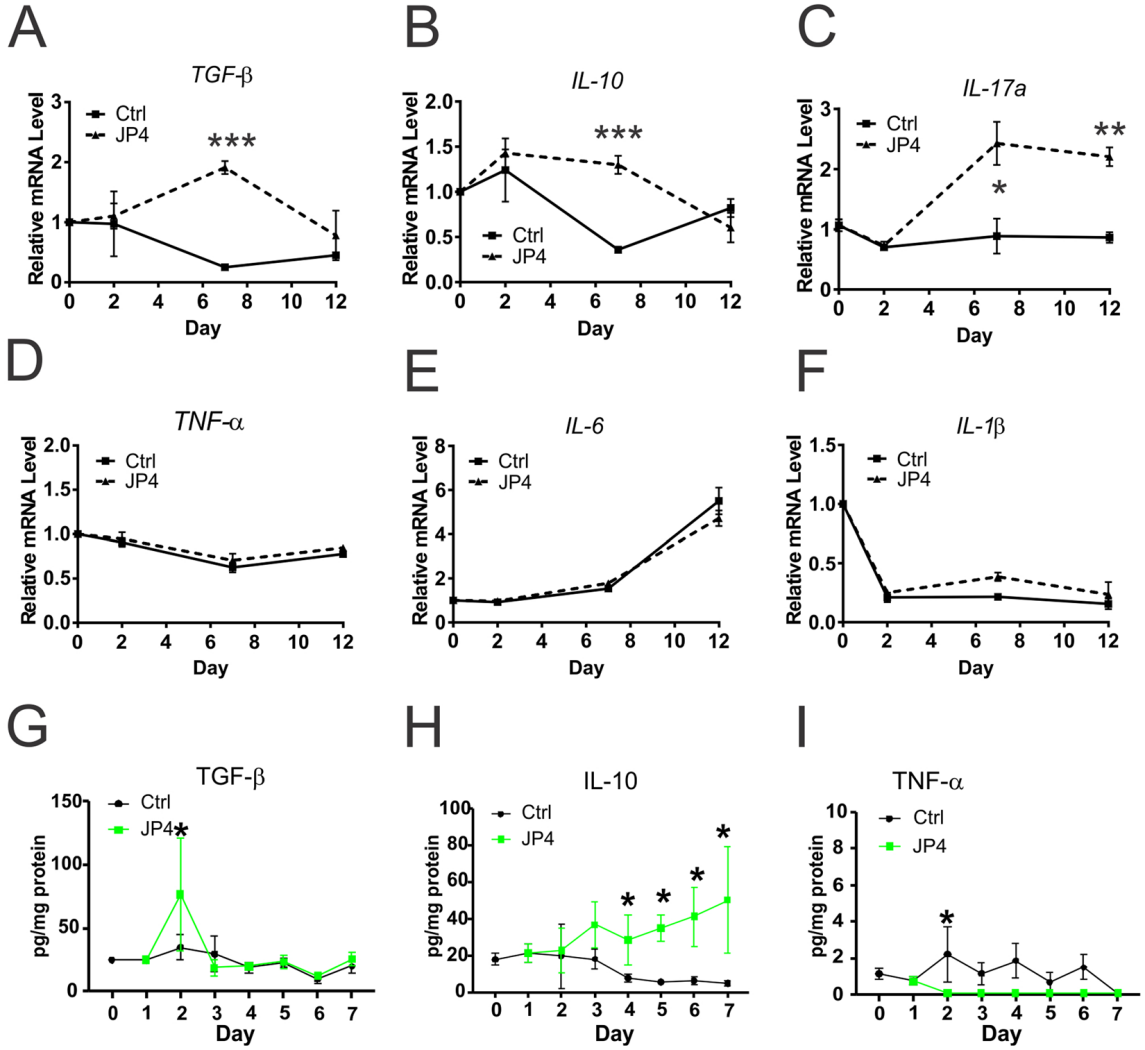
JP4-039 modulates intestinal expression of cytokines after TBI. JP4-039 (JP4, 20 mg/kg in F14 emulsion) or vehicle (Ctrl) was given to mice once 24 hr after 9.25 Gy TBI. Intestinal tissues were analyzed at indicated times (day) after TBI. (**A**–**F**) Intestinal mucosal expression of indicated cytokines. cDNA was synthesized from RNA pooled from 3 mice per group. Expression was normalized to that on Day 0, prior to TBI. n = 3. (**G**–**H**) the cytokine levels of whole intestine. n = 4–5. Values are Mean ± SEM; ***P < 0.001, **P < 0.01. *P < 0.05, vehicle *vs*. JP4, unpaired 2-tailed Student’s t test.

Defective barrier causes immune infiltration and production of cytokines which can further compromise the permeability. We monitored the expression of a panel of cytokines in the intestinal mucosa by qRT-PCR on day 0, 2, 7 and 12 after 9.25 Gy TBI with or without JP4-039. TBI-induced decreases in intestinal protective cytokines such as *TGF-β*^33^, *IL-10*^34^ and *IL-17a*^13^ were blocked by JP4-039 (Fig. 4A–C), but not those prototypic pro-inflammatory cytokines such as *TNF-*α, *IL-6*, and *IL-1*β (Fig. 4D–F). Luminex multiplex assays were used to analyze the levels of 32 cytokines in the intestine, and confirmed increased TGF-β and IL-10 as early as Day 2 in the JP4-039 group, compared to the control, but little or no change in the majority such as TNF-α, IL-1β and IL-6 (Fig. 4G–I, and data not shown). These results demonstrate that TBI induces rapid and profound local changes of inflammatory cytokines, while JP4-039 treatment selectively elevates the expression of several intestinal protective cytokines.

### JP4-039 improves ISC recovery

**Figure 5:**
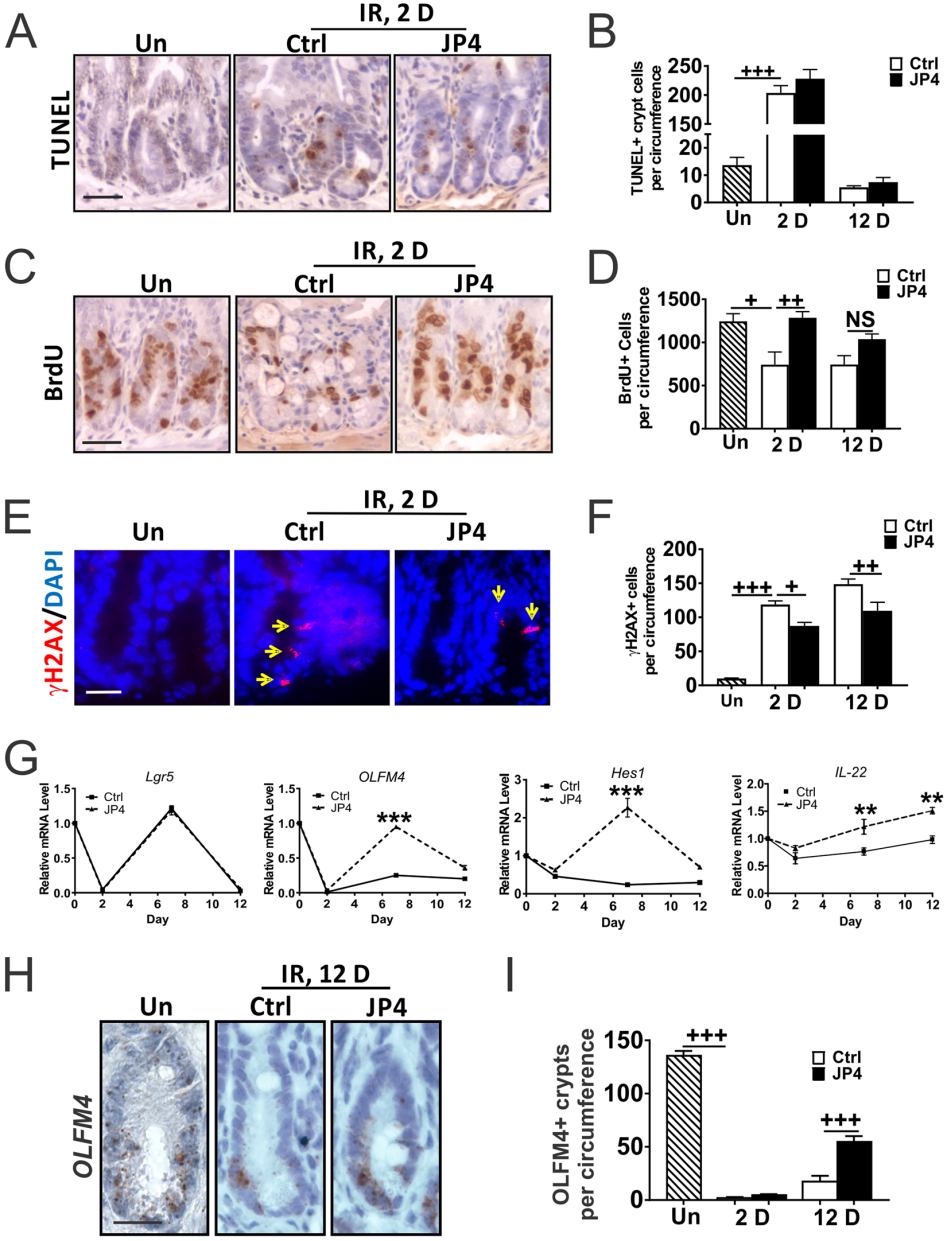
JP4-039 improves DNA repair and ISC recovery after TBI injury. JP4-039 (JP4, 20 mg/kg in F14 emulsion) or vehicle (Ctrl) was given to mice once 24 hr after 9.25 Gy TBI. Intestinal tissues were analyzed at indicated times (day) after TBI. (**A**) Representative TUNEL staining in the crypts. Scale bar = 25 µm. (**B**) Quantification of TUNEL+ crypt cells. (**C**) Representative BrdU staining in the crypts. Scale bar = 25 µm. (**D**) Quantification of BrdU+ crypt cells. (**E**) Representative γH2AX staining in the crypts. Arrows indicate cells with intensive foci that are absent in unirradiated mice. Red, γH2AX, Blue, DAPI. Scale bar = 25 µm. (**F**) Quantification of γH2AX+ crypt cells. (**G**) Mucosal mRNA expression of indicated ISC associated markers. cDNA was synthesized from RNA pooled from 3 mice/group. Expression was normalized to that on Day 0, prior to TBI. ***P < 0.001, **P< 0.01, vehicle *vs*. JP4, unpaired 2-tailed Student’s t test. (**H**) Representative crypt *OLFM4* mRNA staining by RNAscope. Scale bar = 25 µm. (**I**) Quantification of *OLFM4*+ cells in the crypts. *OLFM4*+ crypts were defined as crypts containing 3 or more spots. (**B**,**D**,**F**,**I**), values are Mean ± SEM; n = 3 mice in each group. ^+++^*P* < 0.001, ^++^*P* < 0.01, ^+^*P* < 0.05, 1-way ANOVA followed by Tukey’s multiple comparisons test.

Work from us and others has established that TBI triggers p53 and caspase-dependent crypt apoptosis which peaks within 4–6 hours, and is followed by p53- and caspase-independent cell death after 24 hours independent of the BM injury^22,35,36^. 9.25 Gy TBI induced a 15-fold increase in TUNEL+ cells on Day 2, which returned to the basal level by Day 12 and was unaffected by JP4-039 (Fig. 5A,B). Induction of p53 and targets PUMA and p21, or cell cycle regulators *p15* and *p16* were also unaffected on Day 2 (Fig. S4A–C and data not shown). TBI suppressed crypt proliferation (BrdU) with extensive DNA damage (γH2AX), which was significantly reduced in the JP4-039 group on Day 2 and 12 (Fig. 5C–D). Further, JP4-039 treatment was unable to prolong animal survival or increase crypt regeneration in mice receiving 15 Gy abdominal irradiation (ABI) or TBI (data not shown), where the lethality is caused by ISC depletion and extensive structural damage.

We then analyzed the effects of JP4-039 on ISC markers (*Lgr5, OLFM4*), Wnt (*Wnt 3a, Wnt 2b and Wnt 5a*), BMP (*BMP4, noggin*), EGF, Notch (*OLFM4, Hes1, Math1, Neurogenin 3*, Notch ligand *Dll1)* signaling, and IL-22 on day 0, 2, 7 and 12 after 9.25 Gy TBI. TBI caused strong induction of Wnts and reduction of most other genes as early as by Day 2 (Figs 5G and S5A–F). Interestingly, JP4-039 significantly increased Notch targets *OLFM4, Hes1 and Math 1* on Day 7 and Day 12 (12-fold and 2-fold), and *IL-22* (Figs 5G, S5G–I), both implicated in ISC proliferation and regeneration^10,37,38^. The expression of *OLFM4* in the crypts was confirmed by RNA ISH (Fig. 5H–I). We examined additional ISC and progenitor markers CD44^37^ and Sox9^39,40^ by staining. CD44+ and SOX9+ cells were diminished by Day 12, but unaffected by JP4-039 (Fig. S5J–M). The above findings strongly suggest that JP4-039 given 24 hours after TBI improves the recovery of the intestinal barrier and stem cells likely via Notch and IL-22 signaling.

### Bone marrow transplantation improves intestinal recovery while abnormalities persist in TBI survivors

**Figure 6:**
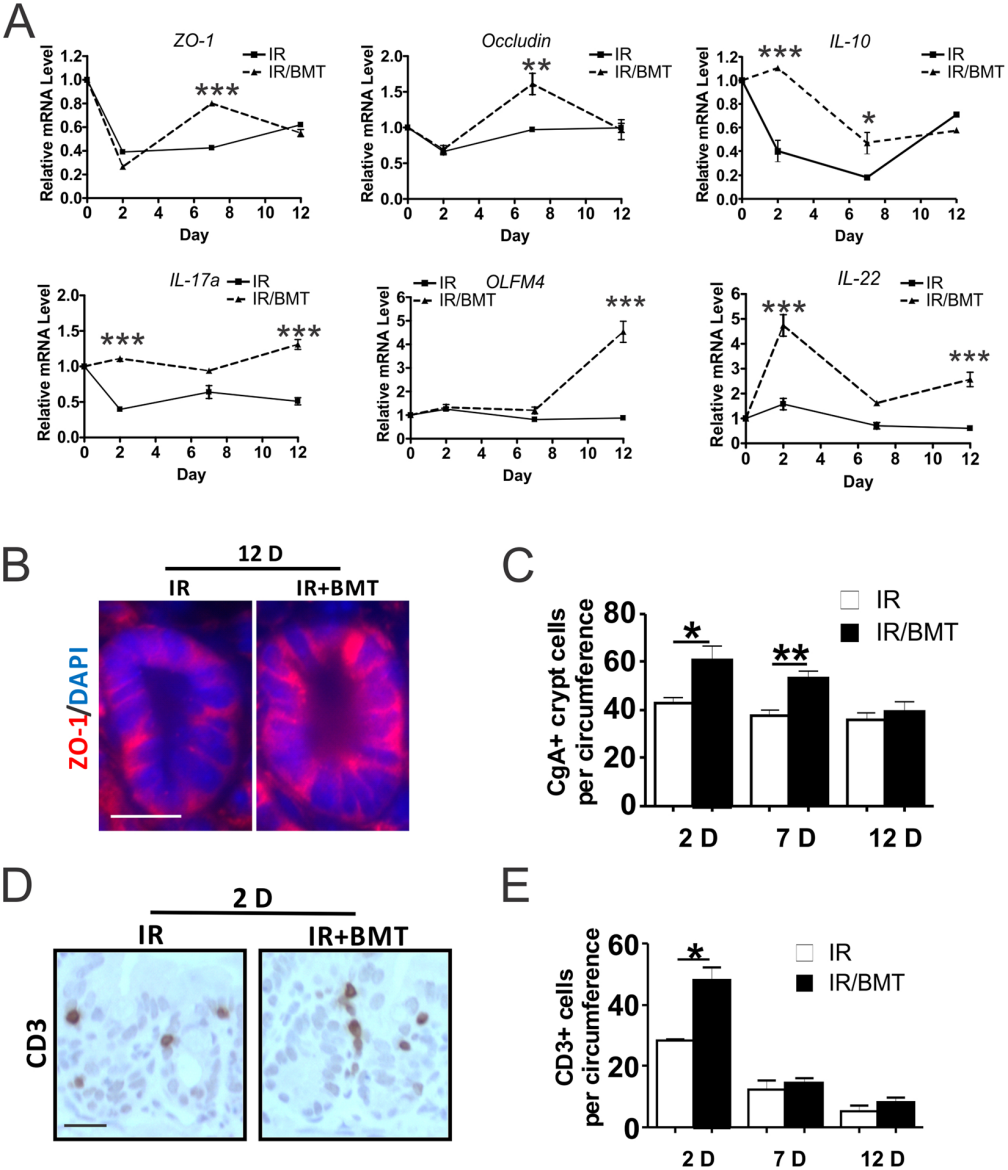
BMT improves intestinal barrier and ISC recovery after TBI. BMT was performed 24 hr after 9.25 Gy TBI. Intestinal tissues were analyzed at indicated times (day) after TBI. (**A**) Intestinal mucosal expression of the indicated genes. cDNA was synthesized from RNA pooled from 3 mice per group. Expression was normalized to that on Day 0, prior to TBI. n = 3. (**B**) Representative immunofluorescence staining of ZO-1 in the crypts. Red, ZO-1; Blue-DAPI. Scale bar = 25 µm. (**C**) Quantification of CgA+ cells in the crypts. (**D**) Representative immunohistochemistry staining of CD3 (T cells) around the crypts. Scale bar = 25 µm. (**E**) Quantification of T cells in (**D**). (**A**,**C**,**E**) values are Mean ± SEM; n = 3 mice in each group. ***P < 0.001, **P < 0.01, *P < 0.05, IR *vs*. IR/BMT, unpaired 2-tailed Student’s t test.

BMT fully rescues lethality of C57BL/6 mice after 9.25–10 Gy TBI (LD30-LD100 in 30 days), and the efficiency declines as the dose goes up with more severe GI injury^15,17^. To test if BMT affects intestinal recovery, we analyzed the structure and major TJ and ISC markers modulated by JP4-039 treatment in mice receiving BMT 24 hours after 9.25 Gy TBI (IR/BMT mice) at several time points (Day 0, 2, 7 and 12). BMT had no effect on intestinal structure by Day 12 (Fig. S6A–B), but modulated most markers, albeit with different kinetics or extent. BMT improved barrier recovery (*ZO-1, Occludin*,) with significant induction of *IL-10, IL-17a, IL-22*, Notch signaling (*OLFM4, Dll1, Math 1, HES1, HES5*), and increased ZO-1 and CgA staining (Figs 6A–C, S6C). No significant induction in *TGF-*β was detected in the BMT mice (Fig. S6C), while a significant increase of T cells around the crypts was detected at Day 2 (Fig. 6D–E). This might contribute to IL-23-independent production of IL-17 to maintain the intestinal barrier^13^.

**Figure 7:**
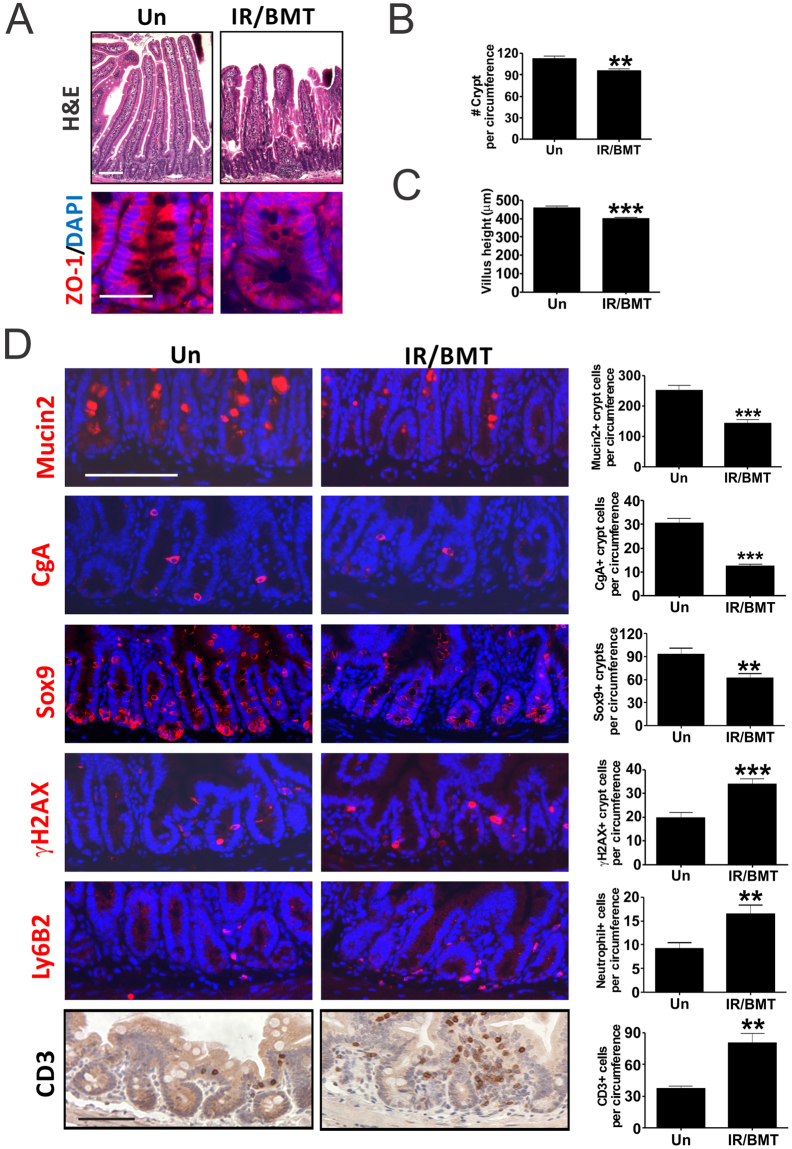
TBI survivors with bone marrow transplantation display persistent intestinal injury with defective barrier and immune imbalance. Female C57BL/6 recipient mice were exposed to 10 Gy TBI followed by bone marrow transplantation (BMT) 24 hr later and recovery of 8 weeks. Intestinal tissues were analyzed in BMT recipients (IR/BMT) and age matched unirradiated (Un) mice. (**A**) Representative H&E staining (upper) and immunofluorescence staining of ZO-1 (bottom) of intestinal sections in the control and IR/BMT mice. Scale bar = 100 µm, or 25 µm (bottom). (**B**) Quantitation of crypt numbers and (**C**) villus height from A. (**D**) Representative immunofluorescence and immunohistochemistry staining of indicated intestinal, DNA damage, and immune markers (left). Scale bar = 100 µm. (Right) Quantification. (**B,C,D**) values are Mean ± SEM; n = 3 mice in each group. ***P < 0.001, **P < 0.01, Un *vs*. IR/BMT, unpaired 2-tailed Student’s t test.

To further determine if GI abnormalities persist in mice saved from the HP syndrome by BMT, we analyzed BMT mice 8 weeks after 10 Gy (LD100/30 days without BMT) TBI (IR/BMT mice). H&E staining indicated minor crypt loss and shortened villi with no change in crypt depth, but a prominent loss of ZO-1 in the crypts in BMT recipients, compared to gender and age-matched, unirradiated control mice (Figs 7A–C, S7A–C). The BMT mice exhibited significant reduction in goblet (Mucin 2+) cells (43%), enteroendocrine (CgA+) cells (60%), the Sox9+ ISC/progenitor compartment (34%), and TUNEL+ crypt cells (40%), with a significant increase in DNA damage (γH2AX+) and immune infiltrates (neutrophils and T cells) (2-fold). No frank inflammation or change in Paneth cells or intestinal proliferation was observed (Figs 7D, S7D–H). Of note, cell death and reduction in the Sox9+ compartment were much lowered compared to day 12 after TBI, suggesting that intestinal recovery potentially facilities the rescue from the acute HP syndrome in BMT recipients. However, persistent barrier defects likely contribute to late effects associated with elevated immunity in TBI survivors due to increased exposure to molecular patterns from microbes (foreign) and damaged cells (self), as well as further impairment of stem cell genomic integrity associated with elevated γH2AX.

## Discussion

Total body irradiation (TBI) initiates a process of “radiation disease,” which begins with the generation of free radicals, DNA strand breaks, loss of stem and progenitor compartments in critical tissues such as the BM and GI tract, and rapidly expands to production of inflammatory cytokines, expression of stress response genes, ultimately leading to multiple organ toxicity and multiple organ failure. Our work strongly supports that central to this pathology is rapid disruption of the intestinal barrier and “irreparable GI damage” leading to ISC dysfunction^21,41^. Impaired ISC self-renewal, differentiation and barrier dysfunction is detected in the acute phase even after 8 Gy TBI (data not shown)^35^, and persists in TBI survivors rescued by BMT. We showed that administration of JP4-039 or BMT 24 hours after TBI significantly improves the barrier and ISC recovery, consistent with GI protective effects by BM or bone marrow stromal cells^21,23–26^. Ligands of Toll-like receptors (TLR2/6, TLR4, TLR5 and TLR9) have been shown to protect against and, to a lesser extent mitigate, radiation-injury^42–45^. It would be interesting to determine if TLR ligands also improve the recovery of the intestinal barrier and ISCs when given as mitigators (i.e., 24 hours after TBI). It has been noted that their efficacy declines significantly if given after TBI, suggesting distinct targets and mechanisms likely involved in radiation protection and mitigation. Since the longevity of stem cell-driven tissues ultimately depends on the genomic integrity and number of cell divisions^46,47^, early intervention in the GI epithelium might significantly impact BM recovery and long-term outcome and survival.

A significant number of surviving mice from TBI eventually develop multiple organ dysfunction syndrome (MODS), and die from sepsis, GI bleeding and multiorgan failure (MOF), similar to human victims of accidental exposure after intensive medical interventions^48,49^. Patients receiving abdominal radiotherapy can develop “leaky gut syndrome” with increased permeability but no frank structural damage or inflammation^50^. TBI survivors rescued by BMT display defective a ISC pool, showing persistent DNA damage and elevated immunity, linked to cellular senescence and production of inflammatory cytokines^51,52^. This important aspect has largely been ignored due to acute GI-driven lethality in high dose TBI models. The major challenge is therefore to identify early modulators and biomarkers predicting long-term outcome of survivors^21,48,49^. Our study demonstrates that TBI induces rapid and profound barrier and ISC dysfunctions. JP4-039 and BMT given 24 hours after TBI significantly improved intestinal recovery on TJs and selective induction of gut protective cytokines IL-10^34^, IL-17a^13,14^. However, extensive changes in ISC markers and signaling pathways induced by TBI and mitigators caution the inherent difficulties of studying stem cells using single marker-based approaches, emphasizing the importance of combining gene signatures and functional assessments in the acute and delayed phases.

A rather surprising finding is that JP4-039 and BMT significantly modulate Notch^38,53^ and IL-22^10^, suggesting common mechanisms involved in intestinal and ISC recovery after TBI. Tissue injury leads to production and release of damage associated molecular patterns (DAMPs), ROS, and inflammatory cytokines that help recruit immune cells to clear damaged cells and initiate remodeling and repair. As an antioxidant, Nitroxide decreases mucosal damage in experimental colitis associated with increased oxidative damage and permeability^27^. Bone marrow stromal cells can suppress inflammation and improve tissue regeneration after injury^21,24^. Interestingly, Notch signaling was recently shown to regulate HSCs after radiation or infection, potentially via redox-sensitive mechanisms^54,55^. It would be interesting to determine if mitigators improve the intestinal barrier and stem cell recovery via ROS and immune-dependent mechanisms^56,57^. Given the highly dynamic and transient nature of IL-22 and Notch signaling during injury and regeneration and their key roles in development and health^33,53^, conditional or pharmacological manipulation is likely required to help better understand their significance and guide the development of new radiation mitigators.

It has become increasingly apparent that the intestinal epithelium plays a key role in human health and disease, and there is a great need and interest to understand the nature of ISC injury, and develop countermeasures to prevent or mitigate acute and delayed gut dysfunction after accidental or medical exposure to radiation. TBI induces complex responses, DNA and oxidative damage, cell death, barrier and local immune and ISC niche perturbation^21,31^. Our work suggests that restoration of the intestinal epithelium and barrier, dictated by ISCs renewing at the fastest rate among all adult tissue, is likely to be crucial for the recovery from radiation injury and reducing long-term complications. Temporal modulation of cytokine and Notch signaling might provide new ways to maximize tissue recovery and to avoid excessive remodeling or cancer risks.

## Materials and Methods

### Mice and Irradiation

The procedures for all animal experiments were approved by the Institutional Animal Care and Use Committee of the University of Pittsburgh. All methods were performed in accordance with the relevant guidelines and regulations. C57BL/6NTac adult (18–20 gram) female mice were irradiated at a rate of 76 cGy/min in a ^137^Cs irradiator (Mark I; JL Shepherd and Associates, San Francisco, CA). Control irradiated mice groups received 9.25 Gy (or 9.5 Gy) total body irradiation. Subgroups received JP4-039 I.V. at 24 hr. Mice were sacrificed at indicated times after TBI for harvesting of intestinal mucosa (N = 3/group) for RNA, protein, or histological analysis.

JP4-039 was synthesized and formulated in F14 emulsion as described^28,29^, and administered I.V. to deliver a dose of 20 mg/kg (approximately 400 µg/mouse) 24 hr after TBI. Bone marrow transplant was performed as described using C57BL/6 mice after lethal 10 Gy TBI^36^, or in C57BL/6NTac after 9.25 TBI for short-term experiments. More details are found in supplemental materials.

### Analysis of mRNA and protein expression

Total RNA and protein were prepared from freshly isolated small intestine as described^35,58^. cDNA were generated and Real-time RT-PCR was performed on CFX96 Touch Real-Time PCR Detection System (Bio-Rad, Hercules, CA) with SYBR Green (Invitrogen). Primers for Real-time PCR are listed in Table S1. Total extracted proteins were subjected to NuPage gel (Invitrogen) electrophoresis followed by Western blotting. Information on antibodies is found in Table S2.

### Tissue processing, Histological Analysis, TUNEL and BrdU staining

Some mice were given 100 mg/kg BrdU by intraperitoneal injection 2 hr prior to sacrifice. Tissues were collected, fixed and embedded in paraffin. Five µm sections were subjected to staining for histological analysis^35,58^. Villus height, crypt depth, and crypt number were measured using H&E stained cross sections^20,36^. Villus height and crypt depth measurements were based on total 50–100 villi or crypts located at different random locations of the jejunum from three mice per group. ImageJ 1.46 software (National Institutes of Health, USA) was used to measure the selected villi or crypts. TUNEL and BrdU staining were performed to evaluate apoptosis and proliferation as described^20,22,35^. All measures were quantified from 3–5 full cross sections in each mouse from three mice per group. More details are found in supplemental materials.

### Immunohistochemistry (IHC) and immunofluorescence (IF)

Paraffin embedded sections were subjected to deparaffinization and antigen retrieval (boiling for 10 min in 0.1 M citrate buffer, pH 6.0, with 1 mM EDTA), followed by staining. Olympus BX51 system microscope equipped with SPOT camera and SPOT Advanced 5.1 software was used to acquire the images. All measures were quantified from 3–5 full cross sections in each mouse from three mice per group. Antibody information is found in Table S2. More details are found in supplemental materials.

### RNA *In Situ* Hybridization

RNA-ISH was performed with RNAscope® 2.0 HD Reagent Kit-Brown (310035 ACD, Hayward, CA) according to manufacturer instructions as described^20^. In briefly, deparaffinized sections were pre-treated with Pretreat 1, 2 and 3. *Olfm4* probe (311831, ACD, Hayward, CA) was added and incubated in the HybEZ oven (310010; ACD, Hayward, CA) for 2 hours at 40 °C. After signals amplification steps, tissue were detected by DAB and counterstained.

### Intestine cytokines levels

Mice were subjected to TBI and indicated treatments and sacrificed with the intestine removed and frozen. We utilized TGFB1 Single Plex Magnetic Bead Kit, as well as a 32 Multiplex Mouse Cytokine/Chemokine Magnetic Bead Panel (EMD Millipore, Billerica, MA, USA) that tested protein concentrations for Eotaxin, G-CSF, GM-CSF, IFN-γ, IL-1α, IL-1β, IL-2, IL-3, IL-4, IL-5, IL-6, IL-7, IL-9, IL-10, IL-12 (p40), IL-12 (p70), IL-13, IL-15, IL-17, IP-10, KC, LIF, LIX, MCP-1, M-CSF, MIG, MIP-1α, MIP-1β, MIP-2, RANTES, TNF-α, and VEGF. For the luminex assay, a sample of 4 mg of intestine was dissected, weighed, and then homogenized in 1 mL of 0.1% Tween 80 in phosphate-buffered saline (PBS) to prevent protein clumping, and stored at −80 °C. Prior to use in the Luminex Protein Assay, intestine homogenate was thawed to room temperature and centrifuged at 2000 rpm at 4 °C for 10 min. Protein concentrations were determined using a BioRad Protein Assay. Samples were plated and prepared for analysis as described by EMD Millipore. The assay was performed on a Luminex Magpix instrument (Luminex, Austin, TX). Data was obtained from 4–5 mice per time point beginning on the day of total body irradiation and daily through Day 7. Data was presented as picogram protein per milligram protein (pg/mg) for intestine. Intestinal homogenate was used to minimize variation in mucosal preparations caused by the extent in damage or regeneration.

### Statistics

GraphPad Prism 7 (GraphPad Software) was used for statistical analyses. Survival was analyzed by the log-rank test. Data were analyzed by an unpaired, 2-tailed Student’s t test or 1-Way ANOVA, followed by Tukey’s test, in which multiple comparisons were performed using the method of least significant difference. A P value of less than 0.05 was considered significant.

## Electronic supplementary material

Supplemental text and Figures
