# Supplementary material for: The GS-nitroxide JP4-039 improves intestinal barrier and stem cell recovery in irradiated mice
Source: Sci Rep. 2018 Feb 1;8:2072. doi: 10.1038/s41598-018-20370-9 (PMC5794877; doi:10.1038/s41598-018-20370-9)

The GS-nitroxide JP4-039 improves intestinal barrier and stem cell recovery in irradiated mice

Liang Wei[1]*, Brian J. Leibowitz[1]*, Michael Epperly [2], Cheng Bi [1, 3], Allen Li [1,4,] Justin Steinman[2], Peter Wipf[3, 5, 6], Song Li[6], Lin Zhang[7], Joel Greenberger[2] and Jian Yu[1, 2#]

[1]Department of Pathology, University of Pittsburgh School of Medicine, UPMC Hillman Cancer Center, PA 15213

[2]Department of Radiation Oncology, UPMC Hillman Cancer Center, PA 15213

[3]Department of Bioengineering, University of Pittsburgh, Swanson School of Engineering

[4]Department of Medicine, University of Pittsburgh, Pittsburgh, PA 15260

[5]Department of Chemistry, University of Pittsburgh, Pittsburgh, PA 15260

[6] Department of Pharmaceutical Science, University of Pittsburgh, Pittsburgh, PA 15260

[7]Department of Pharmacology and Chemical Biology, University of Pittsburgh School of Medicine, Pittsburgh, PA 15213

Running head: JP4-039 mitigates TBI-induced intestinal damage

Key words: radiation injury, Nitroxide, bone marrow transplantation, Notch, TGF-β

*These authors contribute equality to this work.

[#]Correspondence:

Jian Yu, Ph.D., Hillman Cancer Center Research Pavilion, Suite 2.26h, 5117 Centre Ave, Pittsburgh, PA 15213. Email: yuj2@upmc.edu; Phone: 412-623-7786; Fax: 412-623-7778

Supplemental Methods

Mice and Irradiation

JP4-039 was synthesized and formulated in F14 emulsion as described [1, 2], and administered I.V. to deliver a dose of 20 mg/kg (approximately 400 µg/mouse) 24 h after TBI. In brief, JP4-039 (4 mg/ml) + 10% Sesame Oil + 5% Soy Phosphatidyl Choline + 85% Dulbecco's Phosphate-Buffered Saline was placed in ice water with a stream of nitrogen blowing over top. The preparation was sonicated for 1-2 h, and then injected into mice intravenously, at 100 µl containing 400 µg drug or no drug (control) per mouse.

Bone marrow transplant was performed as described [3]. In brief, 7-9 week-old female C57BL/6 mice (The Jackson Laboratory) received 10 Gy total body irradiation the day before receiving transplantation of 1 X 10^6 whole marrow cells from 7-9 week-old male C57BL/6 donor mice. Following transplantation, mice were allowed 8 weeks for recovery and engraftment of transplanted bone marrow. Sex- and age-matched mice without radiation and BMT were used as control for tissue analysis. C57BL/6NTac mice received 9.25 Gy TBI before BMT similarly to match JP4-039 studies.

TUNEL and BrdU staining

TUNEL (Terminal deoxynucleotidyl transferase dUTP nick-end labelling) staining was performed with the ApopTag In Situ Apoptosis Detection Kit (Millipore, Billerica, MA) according to the manufacturer's instructions.

For BrdU staining, sections were deparafinnized and rehydrated through graded ethanols, then treated with Proteinase K (20 µg/ml) for 20 min at 37 °C. Sections were then incubated with 2N HCl for 1 hour at room temperature. Mouse anti-BrdU primary antibody (A21301MP, Invitrogen, Carlsbad, CA; 1:100 in 10% goat serum) and Goat-anti-Mouse-biotin (1:100; #31802; Pierce, Rockford, IL) secondary antibody were used before amplification with the VectaStain ABC kit, and developed with 3,3'-Diaminobenzidine (DAB) (Vector Laboratories, Burlingame, CA).

Immunohistochemistry (IHC) and immunofluorescence (IF)

Mucin2 IF. 20% goat serum was used as blocking solution at room temperature for 1 hour. Sections were incubated overnight at 4 °C with Rabbit-anti-Mucin2 (1:100; sc-15334; Santa Cruz Biotechnology, Santa Cruz, CA). Then the sections were incubated at room temperature for 1 hour with AlexaFluor-594 Goat-anti-Rabbit secondary antibodies (1:100; A11012, Invitrogen) and finally counterstained with VectaShield plus DAPI (Vector Laboratories).

Chromogranin A IF. 20% goat serum was used as blocking solution at room temperature for 1 hour. Sections were incubated overnight at 4°C with Rabbit-anti-Chromogranin A (1:100; ab15160; Abcam, Cambridge, MA). Then the sections were incubated at room temperature for 1 hour with AlexaFluor-594 Goat-anti-Rabbit secondary antibodies (1:100; A11012, Invitrogen) and finally counterstained with VectaShield plus DAPI (Vector Laboratories).

ZO-1 IF. 20% goat serum was used as blocking solution at room temperature for 1 hour. Sections were incubated overnight at 4 °C with Rabbit-anti-ZO-1 (1:100; 61-7300; ThermoFisher, Camarillo, CA). Then the sections were incubated at room temperature for 1 hour with AlexaFluor-594 Goat-anti-Rabbit secondary antibodies (1:100; A11012, Invitrogen) and finally counterstained with VectaShield plus DAPI (Vector Laboratories).

Ly-6B.2 IF/IHC. 20% goat serum was used as blocking solution at room temperature for 1 hour. Sections were incubated overnight at 4 °C with RAT-anti-Ly6B.2 (1:50; MCA771GT; AbD serotec, Hercules, CA). Then the sections were incubated at room temperature for 1 hour with AlexaFluor-594 Goat-anti-Rat secondary antibodies (1:100; A11007, Invitrogen) or biotinylated goat-anti-Rat secondary antibodies (1:100; 31830; Pierce, Rockford, IL) and finally counterstained with VectaShield plus DAPI (Vector Laboratories) or developed with ABC kit and DAB.

γH2AX IF. 20% goat serum was used as blocking solution at room temperature for 1 hour. Sections were incubated overnight at 4 °C with Mouse-anti-γH2AX (1:100; 05-636; Millipore, Billerica, MA). Then the sections were incubated at room temperature for 1 hour with AlexaFluor-594 Goat-anti-Mouse secondary antibodies (1:100; A11005, Invitrogen) and finally counterstained with VectaShield plus DAPI (Vector Laboratories).

CD44 IF. 20% goat serum was used as blocking solution at room temperature for 1 hour. Sections were incubated overnight at 4 °C with RAT-anti-CD44 (1:100; 130012; BioLegend, San Diago, CA). Then the sections were incubated at room temperature for 1 hour with AlexaFluor-594 Goat-anti-Rat secondary antibodies (1:100; A11007, Invitrogen) and finally counterstained with VectaShield plus DAPI (Vector Laboratories).

Sox9 IF. 20% goat serum was used as blocking solution at room temperature for 1 hour. Sections were incubated overnight at 4 °C with Rabbit-anti-Sox9 (1:100; AB5535; Millipore, Billerica, MA). Then the sections were incubated at room temperature for 1 hour with AlexaFluor-594 Goat-anti-Rabbit secondary antibodies (1:100; A11012, Invitrogen) and finally counterstained with VectaShield plus DAPI (Vector Laboratories).

CD3 IHC. 20% goat serum was used as blocking solution at room temperature for 1 hour. Sections were incubated overnight at 4 °C with Rabbit-anti-CD3 (1:100; NB600-1441SS; Novus, Littleton, CO). Then the sections were incubated at room temperature for 1 hour with biotinylated goat-anti-Rabbit secondary antibodies (1:100; 31822; Pierce, Rockford, IL) and finally counterstained with VectaShield plus DAPI (Vector Laboratories) or developed with ABC kit and DAB.

Supplemental Tables

Table S1: qRT-PCR primers used in the study

Gene	Primer	Sequence	Function
BMP4-F	Forward	5'-AGCCCGCTTCTGCAGGA	ISC factor

BMP4-R	Reverse	5'-AAAGGCTCAGAGAAGCTGCG	
Noggin-F	Forward	5'-CGAGCGAGATCAAAGGGCT	ISC factor
Noggin-R	Reverse	5'-TCCTCCTCAGCTTCTTGCTCA	
EGF-F	Forward	5'-AGCATCTCTCGGATTGACCCA	ISC factor
EGF-R	Reverse	5'-CCTGTCCCGTTAAGGAAAACTCT	
Lgr5-F	Forward	5'-GACAATGCTCTCACAGAC	ISC marker
Lgr5-R	Reverse	5'-GGAGTGGATTCTATTATTATGG	
OLFM4-F	Forward	5'-GCCACTTTCCAATTTCAC	ISC marker
OLFM4-R	Reverse	5'-GAGCCTCTTCTCATACAC	
Mucin2-F	Forward	5'-GCCCACCTCACAAGCAGTAT	Goblet cell
Mucin2-R	Reverse	5'-TGCCTTCAGGACAGAAGCAG	
Sucrase Isomaltase-F	Forward	5'-TTGATATCCGGTCCACGGTTCT	Enterocyte
Sucrase Isomaltase-R	Reverse	5'-CAGGTGACATCCAGGTTGCATT	
Claudin-2-F	Forward	5'-TCTACGAGGGACTGTGGATG	tight junction
Claudin-2-R	Reverse	5'-TCAGATTCAGCAAGGAGTCG	
Occludin-F	Forward	5'-GCTGTGATGTGTGTTGAGCT	tight junction
Occludin-R	Reverse	5'-GACGGTCTACCTGGAGGAAC	
ZO-1-F	Forward	5'-ATCAAATCATTACGACCCTG	tight junction
ZO-1-R	Reverse	5'-TGAATGATCTATCCACAGCA	
ZO-2-F	Forward	5'-CCATGGGCGCGGACTATCTGA	tight junction
ZO-2-R	Reverse	5'-CTGTGGCGGGGAGGTTTGACTTG	
ZO-3-F	Forward	5'-AAGCACGCACTCCTGGATGTCACC	tight junction
ZO-3-R	Reverse	5'-GTCGCGCCTGCTGTTGCTGTATTA	
IL-10-F	Forward	5'-ATTTGAATTCCCTGGGTGAGAAG	Cytokine
IL-10-R	Reverse	5'-CACAGGGGAGAAATCGATGACA	
IL-17a	Forward	5'-ATCAGGACGCGCAAACATGA	Cytokine
IL-17a	Reverse	5'-TTGGACACGCTGAGCTTTGA	
IL-1β-F	Forward	5’-ATGGCAACTGTTCCTGAACTCAACT	Cytokine
IL-1β-R	Reverse	5’-CAGGACAGGTATAGATTCTTTCCTTT	
IL-6-F	Forward	5’-AGGATACCACTCCCAACAGACCT	Cytokine
IL-6-R	Reverse	5’-CAAGTGCATCATCGTTGTTCATAC	
IL-22-F	Forward	5'-TTGAGGTGTCCAACTTCCAGCA	Cytokine
IL-22-R	Reverse	5'-AGCCGGACGTCTGTGTTGTTA	
TNFα-F	Forward	5’-TTCTGTCTACTGAACTTCGGGGTGATCGGTCC	Cytokine
TNFα-R	Reverse	5’-GTATGAGATAGCAAATCGGCTGACGGTGTGGG	
TGFβ-F	Forward	5'-CGCCATCTATGAGAAAACC	Cytokine
TGFβ-R	Reverse	5'-GTAACGCCAGGAATTGT	
Dll1-F	Forward	5’-TGAGCCAGTCTTTCCTTGAA	Notch signaling
Dll1-R	Reverse	5’-AGACCCGAAGTGCCTTTGTA	
Hes1-F	Forward	5'-CTCCCGGCATTCCAAGCTAG	Notch signaling
Hes1-R	Reverse	5'-AGCGGGTCACCTCGTTCATG	

Hes5-F	Forward	5'-AGTCCCAAGGAGAAAAACCGA	Notch signaling
Hes5-R	Reverse	5'-GCTGTGTTTCAGGTAGCTGAC	
Math1-F	Forward	5'-ACATCTCCCAGATCCCACAG	Notch signaling
Math1-R	Reverse	5'-GGGCATTTGGTTGTCTCAGT	
Ngn3-F	Forward	5'-CTGCGCATAGCGGACCACAGCTTC	Notch signaling
Ngn3-R	Reverse	5'-CTTCACAAGAAGTCTGAGAACACCAG	
Wnt2b-F	Forward	5'-CACCCGGACTGATCTTGTCT	Wnt signaling
Wnt2b-R	Reverse	5'-TGTTTCTGCACTCCTTGCAC	
Wnt3a-F	Forward	5' CACCACCGTCAGCAACAGCC	Wnt signaling
Wnt3a-R	Reverse	5' AGGAGCGTGTCACTGCGAAAG	
Wnt5a-F	Forward	5'-CACGCTATACCAACTCCTCTGC	Wnt signaling
Wnt5a-R	Reverse	5'-AATATTCCAATGGGCTTCTTCATGGC	
p21-F	Forward	5′-ATGTCCAATCCTGGTGATGT	p53 target
p21-R	Reverse	5′-TGCAGCAGGGCAGAGGAAGT	
PUMA-F	Forward	5'-ATGGCGGACGACCTCAAC	p53 target
PUMA-R	Reverse	5'-AGTCCCATGAAGAGATTGTACATGAC	
GAPDH-F	Forward	5′-CTCTGGAAAGCTGTGGCGTGATG	control
GAPDH-R	Reverse	5′-ATGCCAGTGAGCTTCCCGTTCAG	

**Table S2: Antibodies used in the study**				
**ANTIGEN**	**APPLICATION**	**DILUTION**	**VENDOR**	**CATALOG**
Mucin2	IF	1/100	Santa Cruz	sc-15334
Chromogranin A	IF	1/100	Abcam	ab15160
MMP7	IF	1/50	A&D	AF2967
ZO-1	IF	1/100	ThermoFisher	61-7300
Ly-6B.2	IHC	1/50	AbDserotec	MCA771GT
CD3	IHC	1/100	Novus	NB600-1441SS
Ki-67	IHC	1/100	DAKO	M7249
γH2AX	IF	1/100	Millipore	05-636
CD44	IF	1/100	BioLegend	130012
Sox9	IF	1/100	Millipore	AB5535
Anti-Rabbit Alexa-594	IF	1/100	Invitrogen	A11012
Anti-Rat Alexa-594	IF	1/100	Invitrogen	A11017
Anti-Mouse Alexa-594	IF	1/100	Invitrogen	A11015
Anti-Rabbit Biotin	IHC	1/100	Pierce	31822
Anti-Rat Biotin	IHC	1/100	Pierce	31830
p53	WB	1/1000	Santa Cruz	sc-6243
PUMA	WB	1/1000	Abcam	ab9643
p21	WB	1/1000	Santa Cruz	sc-397
β-actin	WB	1/5000	Sigma	5441
Anti-Rabbit HRP	WB	1/5000	Pierce	31462

Anti-Mouse HRP	WB	1/5000	Pierce	31432

Supplemental references

1. Goff JP*, et al.* Radiobiologic effects of GS-nitroxide (JP4-039) on the hematopoietic syndrome. *In Vivo* **25**, 315-323 (2011).

2. Epperly MW*, et al.* Effectiveness of Analogs of the GS-Nitroxide, JP4-039, as Total Body Irradiation Mitigators. *In Vivo* **31**, 39-43 (2017).

3. Leibowitz BJ*, et al.* Ionizing irradiation induces acute haematopoietic syndrome and gastrointestinal syndrome independently in mice. *Nat Commun* **5**, 3494 (2014).

Supplemental Figure legends

Figure S1. JP4-039 does not alter intestinal structure after TBI. JP4-039 (20 mg/kg in F14 emulsion) or vehicle (Ctrl) was given to mice once 24 hr after 9.25 Gy TBI. Representative H&E staining of small intestine cross sections at Day 0, 2 and 12 after 9.25 Gy TBI. Scale bar = 500 µm.

Figure S2. JP4-039 mitigates abnormal intestinal differentiation after TBI. JP4-039 (20 mg/kg in F14 emulsion) or vehicle (Ctrl) was given to mice once 24 hr after 9.25 Gy TBI. Intestinal tissues were analyzed at indicated times (day) after TBI. (A) Representative immunofluorescence of Mucin 2, and (B) CgA (Chromogranin A) in the crypts. Red, Mucin2/CgA; Blue, DAPI. Scale bar = 100 µm. (C) Representative immunofluorescence of MMP7 in the crypts with indicated treatment. Red, MMP7; Blue, DAPI. Scale bar = 100 µm. (D)

Quantification of MMP7+ crypt cells from C. $^{+}P < 0.05$, 1-way ANOVA followed by Turkey's multiple comparisons test.

Figure S3. JP4-039 maintains the intestinal barrier after TBI. JP4-039 (JP4, 20 mg/kg in F14 emulsion) or vehicle (Ctrl) was given to mice once 24 hr after 9.25 Gy TBI. Intestinal tissues were analyzed at indicated times (day) after TBI. (A) Mucosal mRNA expression of *ZO-3* and *Claudin-2*. cDNA was synthesized from RNA pooled from 3 mice/group. Expression was normalized to that on Day 0, prior to TBI. Values are Mean ± SEM; n = 3 mice in each group. $^{**}P < 0.01$, $^{*}P < 0.05$, vehicle *vs.* JP4, unpaired 2-tailed Student's t test. (B) Representative immunofluorescence staining of ZO-1. Red, ZO-1; Blue-DAPI. Scale bar = 25 µm. (C) Representative immunofluorescence staining of ZO-1. Crypt areas are circled in yellow. Red, ZO-1; Blue-DAPI. Scale bar = 100 µm. (D) Zoomed villus ZO-1 staining, which was unaffected by TBI or JP4-039. Scale bar = 25 µm.

Figure S4. The effects of JP4-039 on the p53 pathway. JP4-039 (JP4, 20 mg/kg in F14 emulsion) or vehicle (Ctrl) was given to mice once 24 hr after 9.25 Gy TBI. Intestinal tissues were analyzed at the indicated time (day 2) after TBI. (A) Mucosal mRNA expression of *PUMA* and (B) *p21*. cDNA was synthesized from RNA pooled from 3 mice per group. Expression was normalized to that on Day 0, prior to TBI. (C) Mucosal expression of indicated proteins analyzed by western blotting. β-actin was used as the loading control. Lysates were pooled from 3 mice/group.

Figure S5. The effects of JP4-039 on intestinal stem cells and signaling. JP4-039 (20 mg/kg in F14 emulsion) or vehicle (Ctrl) was given to mice once 24 hr after 9.25 Gy TBI. Intestinal tissues were analyzed at indicated times (day) after TBI. (A-I) Mucosal mRNA expression of markers in Wnt (A-C), BMP and EGF (D-F) and Notch (G-I) signaling. cDNA was synthesized from RNA pooled from 3 mice/group. Expression was normalized to that on Day 0, prior to TBI. (A-I) *P < 0.05, vehicle *vs.* JP4, unpaired 2-tailed Student's t test. (A, C, E, G) values are Mean ± SEM; n = 3 mice in each group. (J) Representative immunofluorescence of CD44 (Red) and (K) Sox 9 (Red) in the crypts. Blue, DAPI. Scale bar = 100 µm. (L) Quantification of CD44+ and (M) Sox9+ crypt cells. $^{+++}P < 0.001$, 1-way ANOVA followed by Turkey's multiple comparisons test.

Figure S6. BMT modulates intestinal recovery after TBI. BMT was performed in mice 24 hr after 9.25 Gy TBI. Intestinal tissues were analyzed at indicated times after TBI. (A) Representative H&E staining of intestinal sections on Day 12 after TBI. Scale bar = 100 µm. (B) Quantification of crypt numbers, crypt depth, and villus length. Values are Mean ± SEM; n = 3 mice in each group. $^{++}P < 0.01$, 1-way ANOVA followed by Tukey's multiple comparisons test. (C) Mucosal mRNA expression of indicated markers. cDNA was synthesized from RNA pooled from 3 mice/group. Expression was normalized to that on Day 0, prior to TBI. *P < 0.05, **P < 0.001, R *vs.* IR/BMT, unpaired 2-tailed Student's t test.

Figure S7. Persistent GI abnormalities in BMT TBI survivors. Female C57BL/6 recipient mice were exposed to 10 Gy TBI followed by bone marrow transplantation (BMT) at 24 hr and recovery of 8 weeks. Intestinal tissues were analyzed in BMT recipients (IR/BMT) and age-

matched unirradiated (Un) mice. (A) H&E staining of small intestine cross sections. Scale bar = 500 µm. (B) Quantification of crypt depth from A. (C) Representative immunofluorescence of ZO-1. Crypt areas are circled depicting reduced signals compared with the control. Red, ZO-1; Blue, DAPI. Scale bar = 100 µm. (D) Zoomed images of crypt cells with intensive γH2AX foci. Scale bar = 25 µm. (E) Quantification of MMP7+ cells at +4 position or below (lower), (F) at the TA zone (upper), (G) Ki-67+, and (H) TUNEL+ crypt cells. (B, E-H), values are Mean ± SEM; n = 3 mice in each group. $*P < 0.05$, unpaired 2-tailed Student's t test.

Fig S1 related to Fig 1

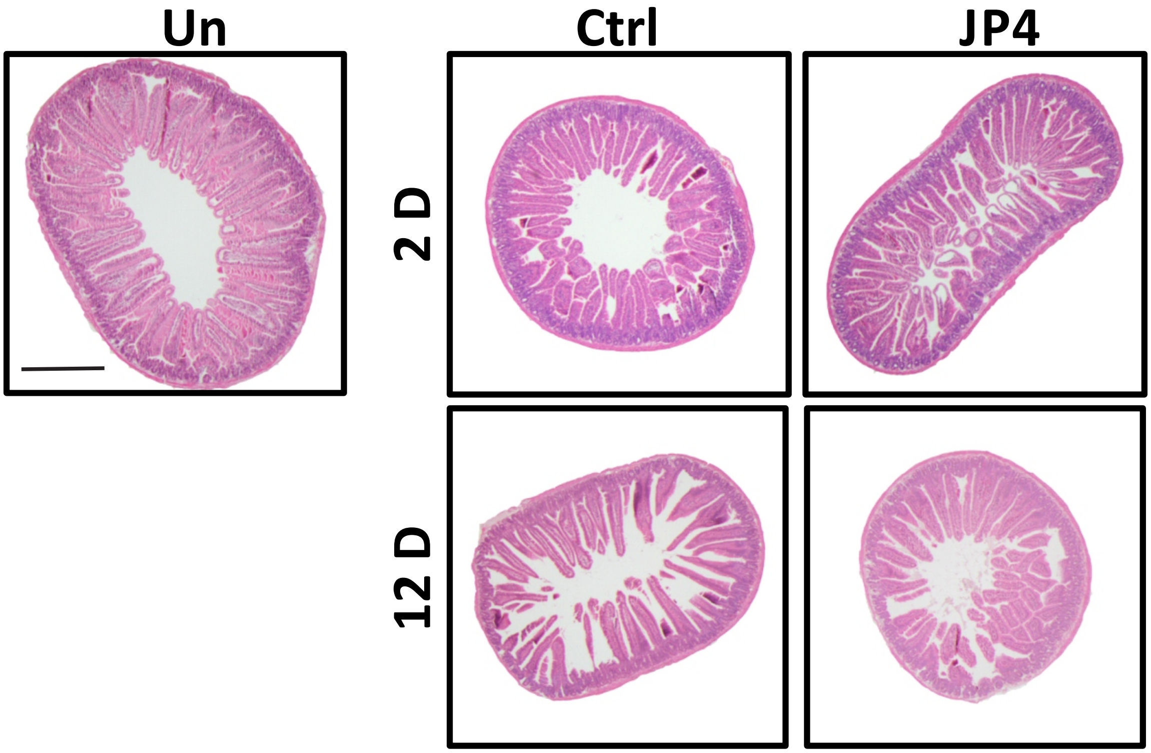

Fig S2 related to Fig 2

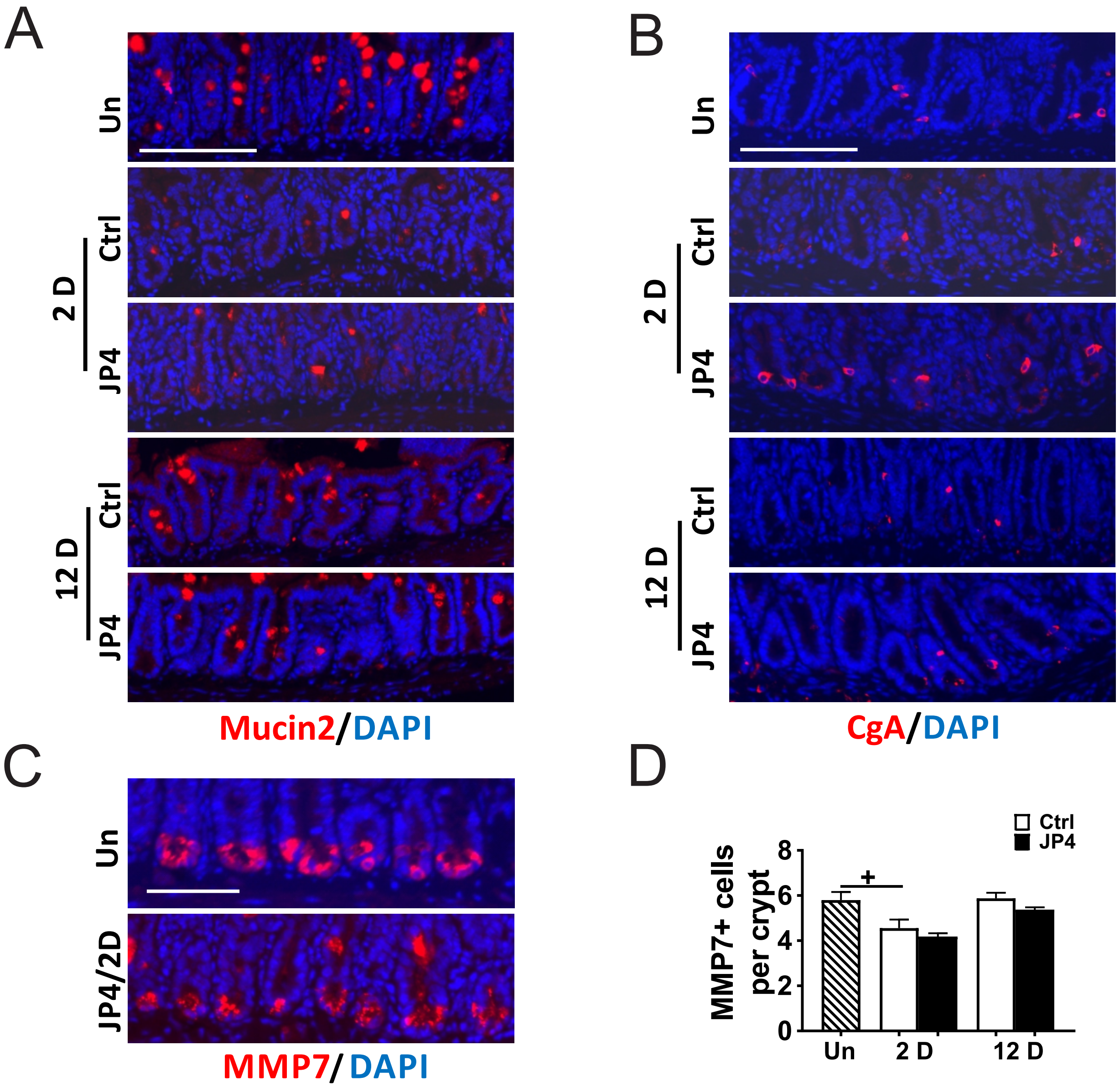

Fig S3 related to Fig 3

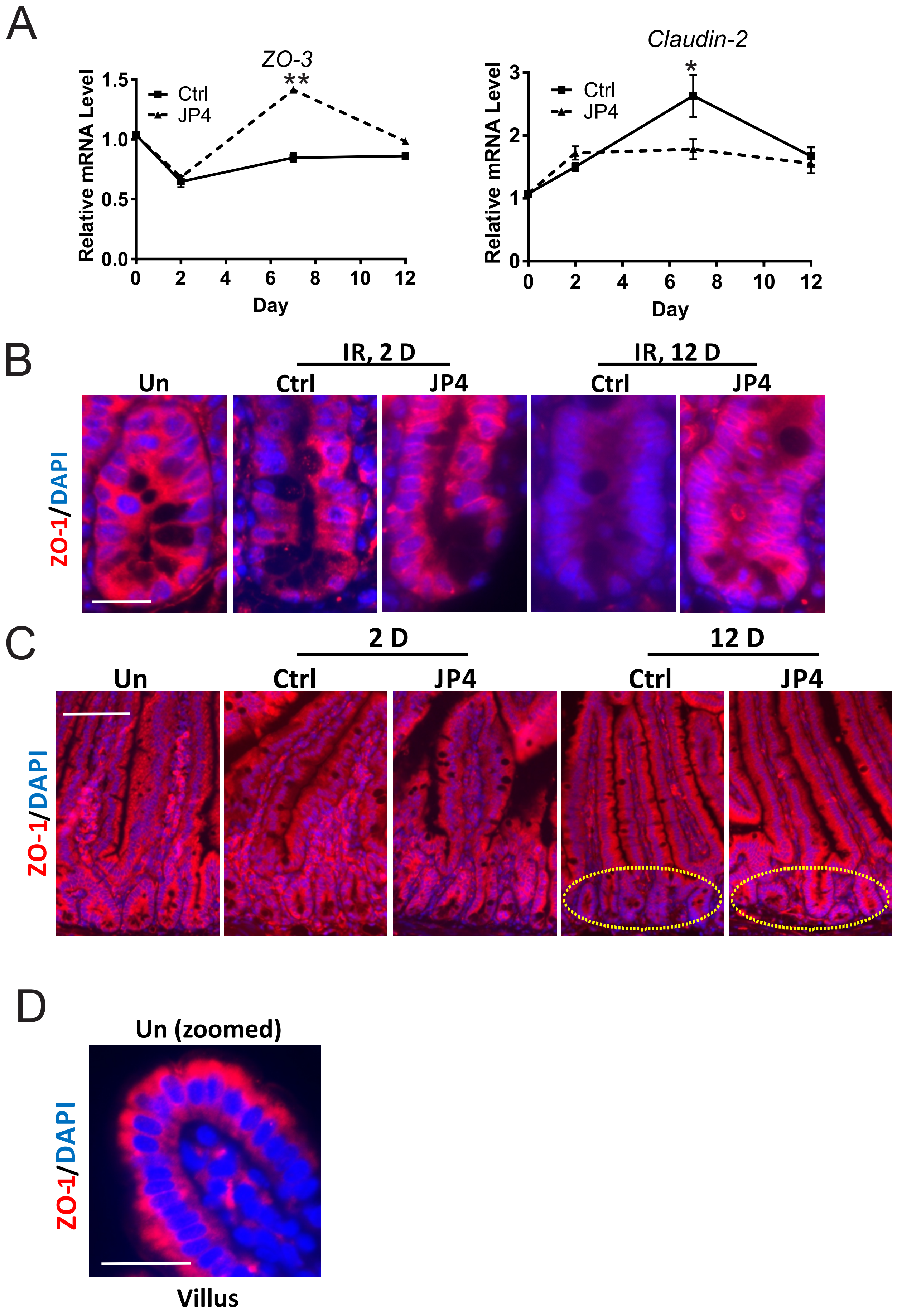

Fig S4 related to Fig 5

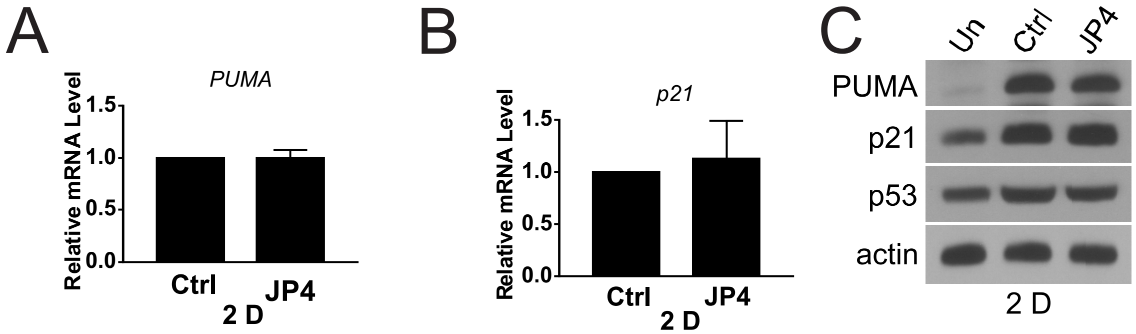

Fig S5 related to Fig 5

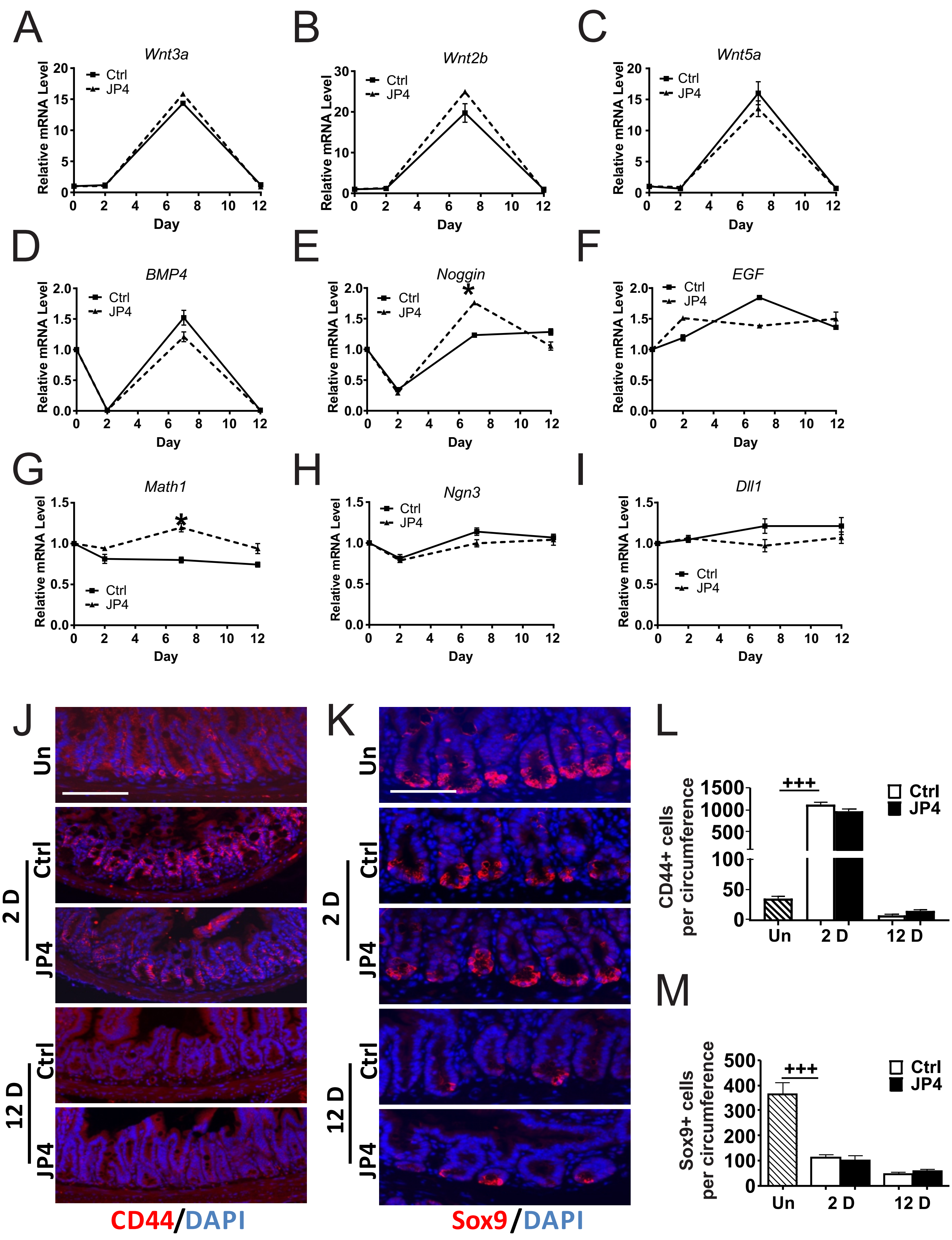

Fig S6 related to Fig 6

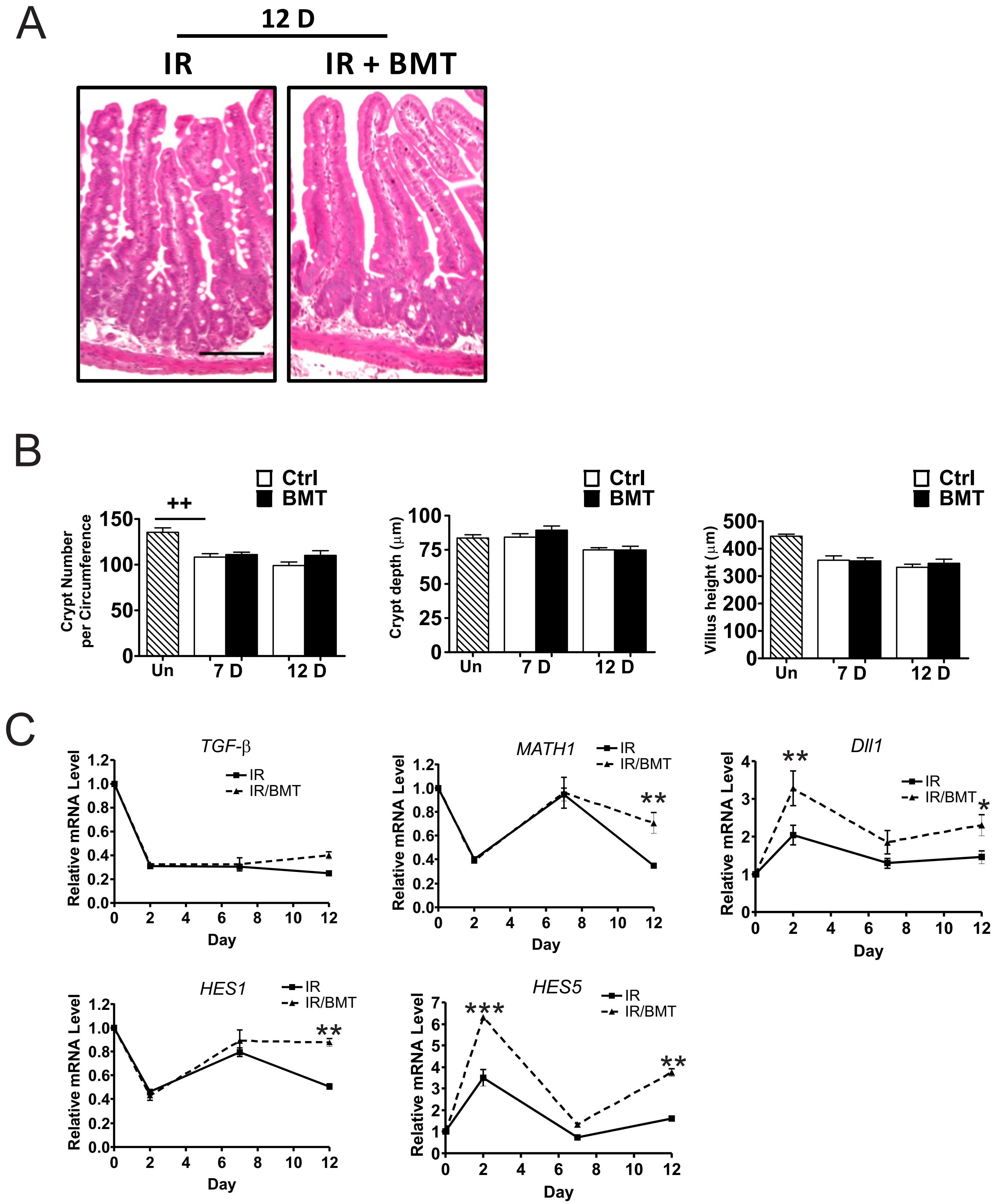

Fig S7 related to Fig 7

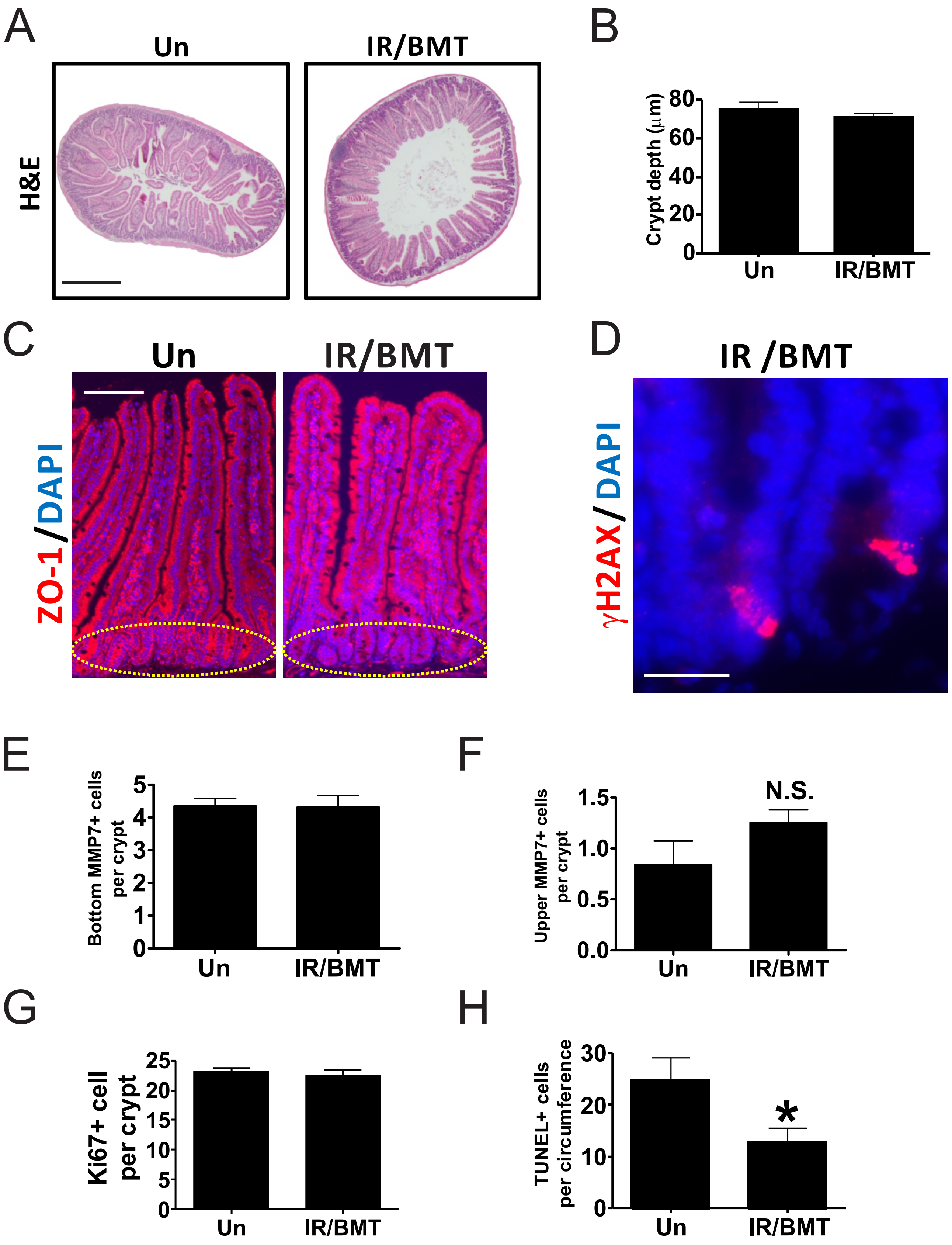

Supplement: Supplementary file 1 — Supplemental text and Figures [file 41598_2018_20370_MOESM1_ESM.pdf]
